# Bright Infrared Colloidal PbS Nanoplatelets with Lead Sulfobromide Shells

**DOI:** 10.1021/acs.chemmater.5c02480

**Published:** 2026-01-19

**Authors:** Sabin Aryal, Yiteng Tang, Dulanjan Harankahage, Mikhail Zamkov, Liangfeng Sun

**Affiliations:** † Department of Physics and Astronomy, 1888 Bowling Green State University, Bowling Green, Ohio 43403, United States; ‡ Center for Photochemical Sciences, Bowling Green State University, Bowling Green, Ohio 43403, United States

## Abstract

We report colloidal PbS nanoplatelets synthesized in bromide-containing media that spontaneously form core/shell heterostructures with inorganic lead sulfobromide and lead bromide shells surrounding a PbS core. Band-structure measurements indicate a type-I alignment, which concentrates electrons and holes within the PbS core. These core–shell nanoplatelets exhibit near-infrared emission with a remarkably narrow photoluminescence peak of 78 meV at room temperature. The inorganic shells also act as robust passivating layers that suppress surface defect states, yielding an improved photoluminescence quantum yield and preserving emissive performance under ambient conditions. The resulting nanoplatelets are bright and air-stable, offering a solution-processable platform for infrared photonics and optoelectronics, including emitters, detectors, and integrated photonic components.

## Graphical abstract

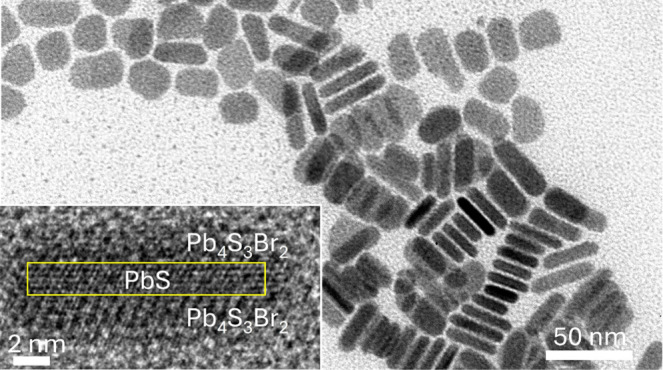

## Introduction

Colloidal two-dimensional (2D) semiconductors, commonly termed nanosheets or nanoplatelets, provide tunable band gaps through control of thickness and adjustable exciton dynamics through variation of lateral dimensions. This structural tunability enables distinctive photonic and optoelectronic phenomena, including low-threshold lasing,^1–3^ giant oscillator–strength transitions,^4,5^ and multiple exciton generation.^6^ However, the large surface area inherent to nanoplatelets can host numerous surface defect states relative to quantum dots, often resulting in exciton quenching. Organic electron-donating (L-type, e.g., phosphines and amines) and electron-accepting (Z-type, e.g., metal carboxylates) ligands^7–10^ can passivate these states and enhance optical responses, but they provide limited stability under ambient conditions and impede charge injection in assembled structures, thereby restricting device integration. In contrast, thin inorganic shells offer more robust passivation, improving environmental stability while maintaining or enhancing optoelectronic performance.^11–16^

Here, we report a facile and robust single-pot synthesis of colloidal PbS nanoplatelets encapsulated by a sulfobromide shell. In this approach, PbBr_2_ serves as a critical lead precursor, directing the 2D growth of the nanoplatelets. The resulting structures typically consist of an ∼1.5 nm PbS core surrounded by an ∼2 nm shell. These core/shell nanoplatelets exhibit bright near-infrared emission with photoluminescence quantum yields up to 35%, an order of magnitude higher than unshelled PbS nanoplatelets. Their photoluminescence decay is nearly single-exponential, with lifetimes exceeding 1 μs. Cyclic voltammetry confirms a type-I band alignment between the PbS core and the sulfobromide shell, ensuring strong exciton confinement and enabling the enhanced optical performance required for optoelectronic applications.

## Results and Discussion

In the synthesis of PbS nanosheets^17–19^ and nanoribbons,^7^ lead oleate and thioacetamide are typically employed as lead and sulfur precursors, respectively. Lead oleate is prepared by reacting lead oxide (PbO) with oleic acid, while a chloroalkane cosolvent (e.g., chloroform) is often introduced to promote 2D attachment of nanocrystals, yielding nanosheets.^7,17–22^ Our recent work demonstrated that chloride ions can also trigger 2D attachment, leading to the formation of PbS nanoplatelets.^23^ Inspired by this finding, we used a combination of PbO and PbBr_2_ as lead precursors (Supporting Information A) and introduced oleylamine^24^ to enhance the solubility of PbBr_2_ in the reaction mixture. This approach produced well-defined 2D nanoplatelets (Figure 1b) with rectangular morphology, exhibiting an average length of 19.3 nm and a width of 11.0 nm, as determined by transmission electron microscopy (TEM) (Supporting Information B). Vertically oriented nanoplatelets reveal a thickness of ∼6.2 nm.

**Figure 1:**
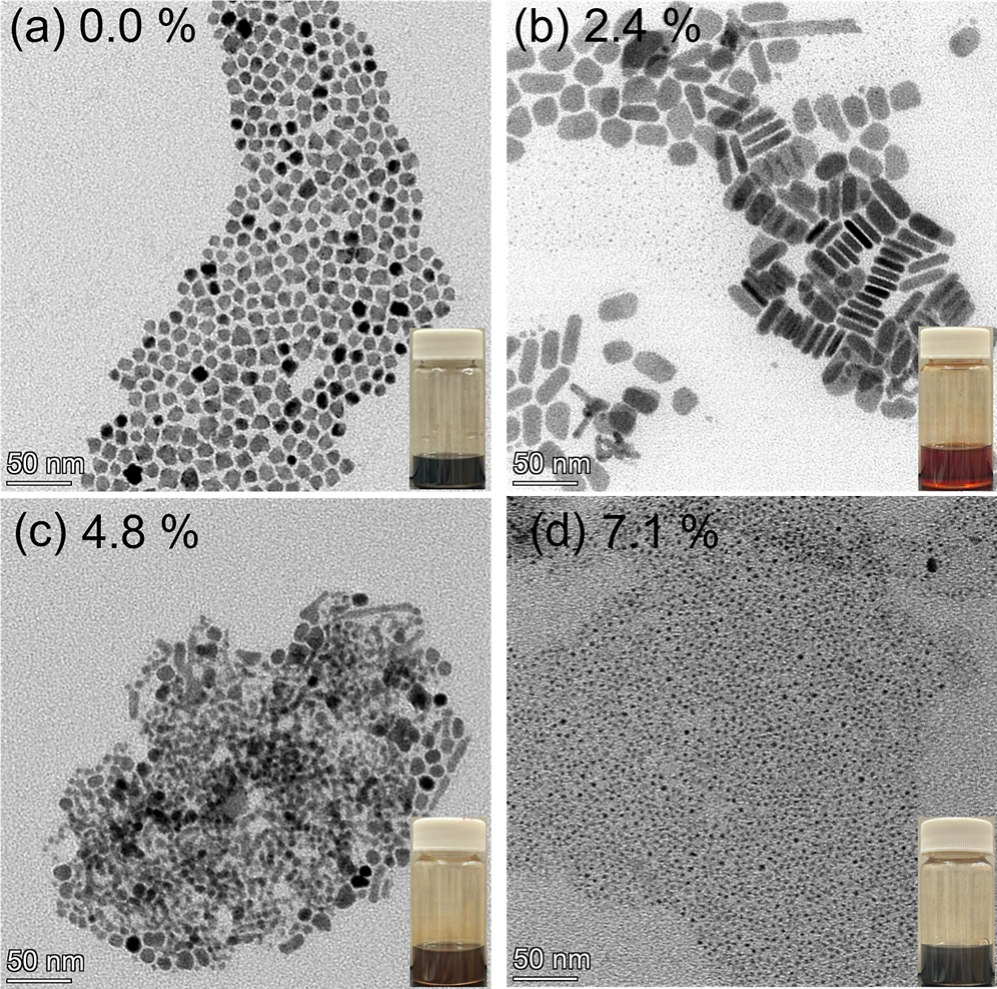
TEM images of nanoplatelets synthesized with varying molar percentages of PbBr_2_ in the lead precursor mixture (PbO and PbBr_2_): (a) 0.0%, (b) 2.4%, (c) 4.8%, and (d) 7.1%. Insets: photographs of the corresponding nanoplatelet–toluene solutions, displaying distinct colors.

Bromide ions play a critical role in directing the 2D growth. To elucidate their effect, we systematically varied the molar ratio of PbBr_2_ to PbO while keeping the total lead precursor concentration constant. In the absence of PbBr_2_, only large quantum dots formed (Figure 1a). At ∼2.4 mol % PbBr_2_, uniform nanoplatelets were obtained (Figure 1b). Increasing the PbBr_2_ fraction to 4.8 mol % yielded irregular nanoplatelets alongside quantum dots (Figure 1c), while further increases produced only small quantum dots (Figure 1d). This trend resembles our previous observations for PbS/PbCl_2_ core/shell nanoplatelets.^23^ We attribute this behavior to the ability of bromide ions to form Pb–Br–Pb bridges that promote oriented attachment of PbS nanocrystals^25–27^ along the (110) facet,^28^ enabling 2D growth. However, at high Br^–^ concentrations, excess passivation of quantum dot surfaces inhibits attachment, favoring isolated dots. Oleylamine, used to dissolve PbBr_2_, may also assist 2D growth by selectively blocking growth along the (100) direction.^28^ The interplay between oleylamine and oleic acid has been shown to strongly influence nanocrystal morphology,^29–31^ suggesting that further tuning of ligand concentrations may provide additional control over nanoplatelet shape.

We employed high-resolution TEM (HRTEM) to verify the core–shell structure of the nanoplatelets (Figure 2a). Depending on their orientation, some nanoplatelets were observed with their basal planes facing upward, while others were oriented vertically on their edges. In the basal-plane orientation, the HRTEM image shows a uniform square lattice (Figure 2b), and the corresponding fast Fourier transform (FFT) pattern reveals a periodicity of 0.29 nm in two orthogonal directions, consistent with the PbS (100) plane. In contrast, the HRTEM image of edge-oriented nanoplatelets reveals a distinctly different crystal structure (Figure 2c). The central layer (core), approximately 1.5 nm thick, displays an ordered atomic array, whereas the top and bottom layers (shell) exhibit a different structural pattern. The atomic arrangement in the core closely resembles that of cubic PbS (galena) viewed along the ⟨110⟩ direction, with lattice fringes forming a 55° angle and showing a periodicity of 0.42 nm along the edge (Figure 2d). The shell also shows a 0.42 nm periodicity along the edge, but its FFT pattern differs significantly from that of the core (Figure 2e), indicating distinct crystallographic phases between the core and shell.

**Figure 2:**
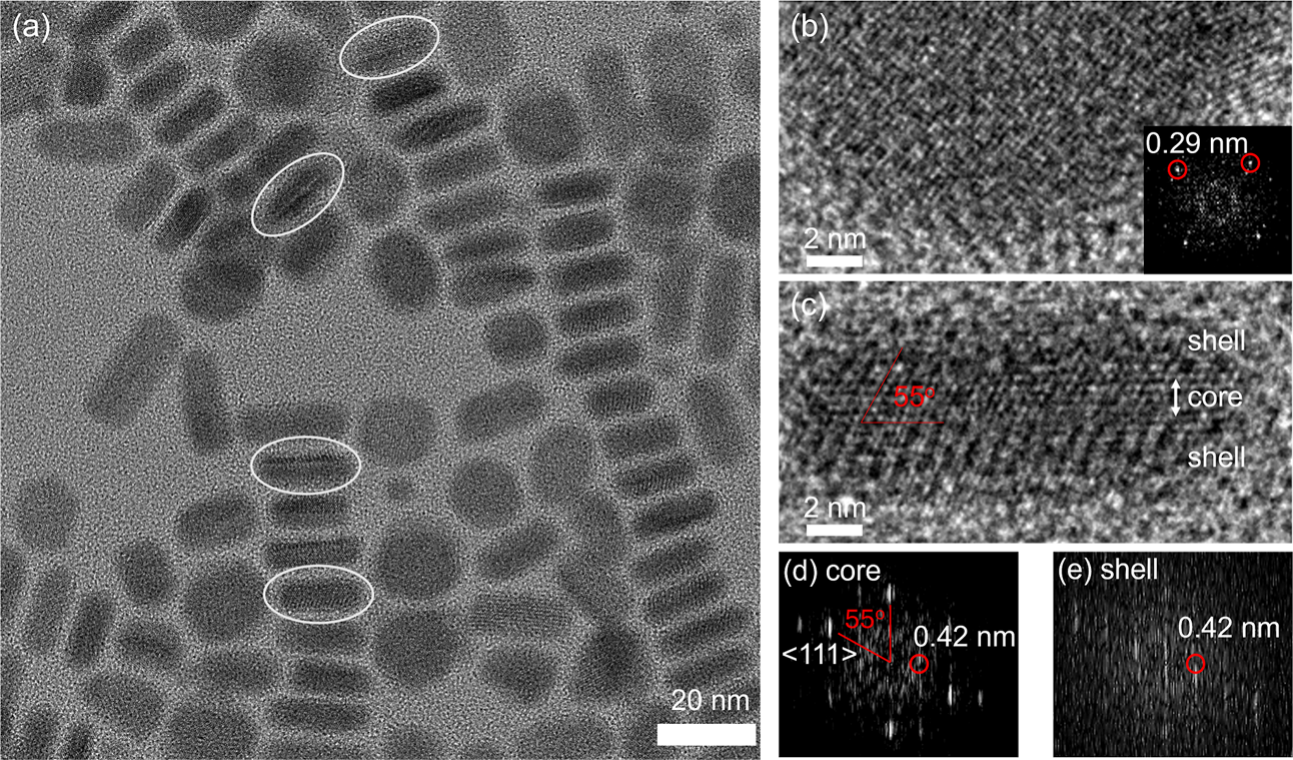
(a) HRTEM image of nanoplatelets synthesized at 120 °C for 20 min. Some nanoplatelets are oriented with their basal planes facing upward, while others are oriented with their edge facets upward. Core/shell structures can be identified in the circled nanoplatelets. (b) Nanoplatelet with its basal plane facing upward. Inset: fast Fourier transform (FFT) image showing a periodicity of 0.29 nm along two orthogonal directions. (c) Nanoplatelet with its edge facet facing upward, revealing distinct crystalline phases of the core and shell. (d, e) FFT images corresponding to the core and shell regions in (c), respectively.

The core/shell structure of the nanoplatelets is further confirmed using X-ray diffraction (XRD). The XRD pattern of the nanoplatelets shows peaks at 30.02°, 42.74°, and 50.21° and a shoulder at 26.09° (Figure 3a). They are close to the reflection peaks from the (200), (220), (311), and (111) crystal planes of cubic galena, respectively. These peaks match well to the XRD peaks from colloidal PbS quantum dots synthesized in our lab, confirming the existence of the PbS crystalline phase in nanoplatelets.

**Figure 3:**
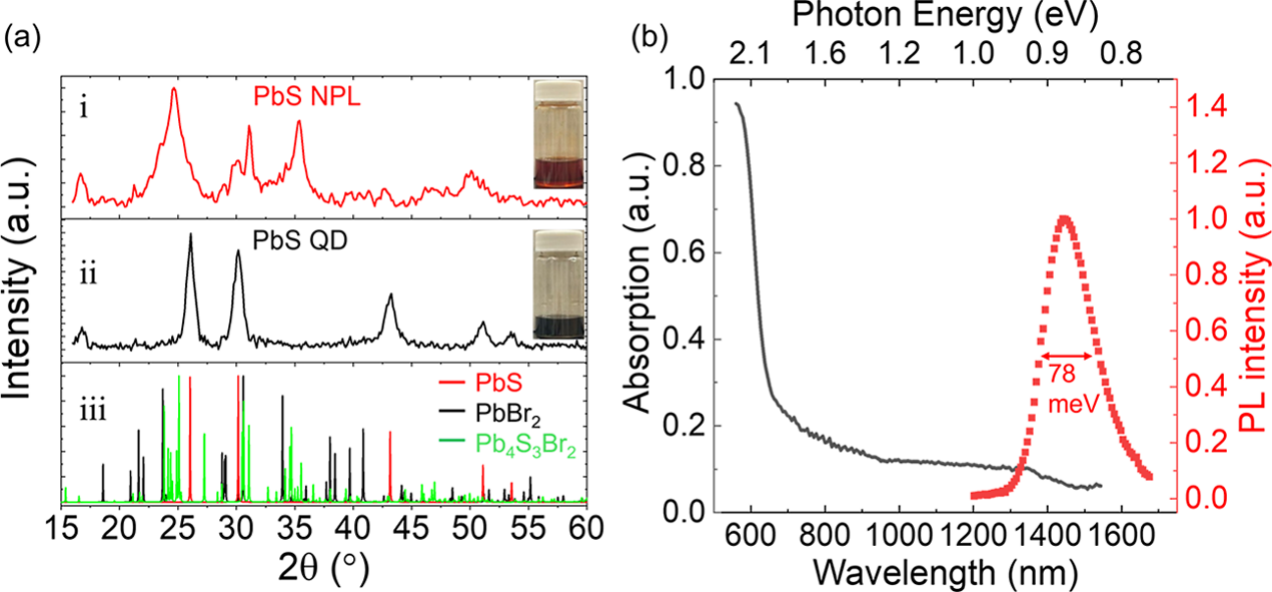
(a) XRD patterns of (i) PbS nanoplatelets (PbS NPL) and (ii) PbS quantum dots (PbS QD), along with (iii) simulated XRD patterns of PbS, PbBr_2_, and Pb_4_S_3_Br_2_. (b) Optical absorption spectrum (solid line) and photoluminescence (PL) spectrum (solid squares) of nanoplatelets synthesized with a growth time of 20 min.

The XRD shoulders at 23.4° and 34.2°, a weak XRD peak at 29.6°, and the peaks between 38° and 41° indicate the existence of PbBr_2_. Their weak intensities indicate a low fraction of the PbBr_2_ crystalline phase in the nanoplatelet. The calculated PbBr_2_ XRD peak corresponding to its (121) plane (*a* = 9.466 Å, *b* = 8.068 Å, *c* = 4.767 Å)^32^ appears at 30.5°, which is close to the PbS (200) peak. The observed peaks at 24.6°, 31.1°, and 35.4° are strong but do not exist in either the XRD of PbS or the XRD of PbBr_2_. These peaks are likely from lead sulfobromide alloys, including Pb_4_S_3_Br_2_ as reported by Imran^33^ and Toso^34,35^ et al. and Pb_3_S_2_Br_2_ as reported by Liu et al.^36^ These two sulfobromide alloys exhibit similar XRD patterns. In the following analysis, we use the Pb_4_S_3_Br_2_ crystal information file (*a* = 8.178 Å, *b* = 14.732 Å, *c* = 8.095 Å)^34^ that is available and shared. We find that the corresponding crystalline planes are (022) at 25.1°, (202) at 31.1°, and (311) at 35.2°.

On the other hand, optical spectroscopy (Supporting Information C) reveals the absorption edge and emission peak, which can be used to estimate the thickness of the PbS cores. Unlike zero-dimensional quantum dots that display sharp excitonic peaks,^37^ two-dimensional nanoplatelets exhibit step-like features in their absorption spectra (Figure 3b).^38–40^ Both the optical absorption edge and emission peak are in the near-infrared region, beyond 1200 nm (Figure 3b). This indicates that the nanoplatelets possess a narrow energy gap, likely originating from PbS cores with a 2D structure similar to PbS nanosheets or nanoribbons. The lead sulfobromide (e.g., Pb_4_S_3_Br_2_ ^33,35^ or Pb_3_S_2_Br_2_ ^36^) shell, which has an energy gap of 1.8 eV and above, unlikely leads to infrared absorption and emission. Since nanoplatelets have a lateral dimension (width or length) close to the Bohr radius of PbS (∼18 nm)^41^ and a thickness much smaller, we expect a loose lateral quantum confinement but a tight quantum confinement in thickness direction. We use the empirical quantum confinement model developed earlier for the PbS nanoribbons^7^ that have a close dimension as the nanoplatelets to derive the thickness of the PbS core.

The nanoplatelets of 20 min growth time show a narrow photoluminescence peak of 78 meV width at 1440 nm and an absorption edge close to it (Figure 3b). Based on the quantum confinement model,^42^ the thickness of the PbS is estimated to be 1.8 nm (Supporting Information D). It is larger than 1.5 nm as measured by HRTEM (Figure 2c). This discrepancy indicates the effect of the alloy shell which might have a band structure close to that of PbS. In consequence, the wave functions of the charge carriers (electrons and holes) in PbS core “leak” into the alloy shell, reducing the quantum confinement. To achieve the same energy gap, a thinner PbS core is needed.

With XRD and optical spectroscopy, we revealed the dynamics of core/shell growth. The XRD patterns of the products at different growth times demonstrate the change of the relative intensity of the XRD peaks corresponding to different crystal phases (Figure 4a). The peak intensity ratio of PbS (200) at 30.4° to Pb_4_S_3_Br_2_ (202) at 31.1° decreases as the growth time increases from 5 to 10, 15, and 20 min (Figure 4b). It indicates the relative decrease of the PbS crystalline phase and the increase of the Pb_4_S_3_Br_2_ crystalline phase in the core/shell structure. The peak intensity ratio of PbS (200) at 30.4° to Pb_4_S_3_Br_2_ (311) at 35.2° exhibits the same trend. Both ratios increase slightly for the 35 min sample, which we attribute to experimental uncertainty arising from its noisier XRD data. The relative increase of the peak at 30.2° might be due to the contribution of PbBr_2_ which has a strong XRD peak at 30.5°, corresponding to its (121) plane. The relative increase of the intensities near 23.7° and 34.0° might also indicate the increase of the PbBr_2_ crystalline phase that has its (301) and (111) peaks at their diffraction angles.

**Figure 4:**
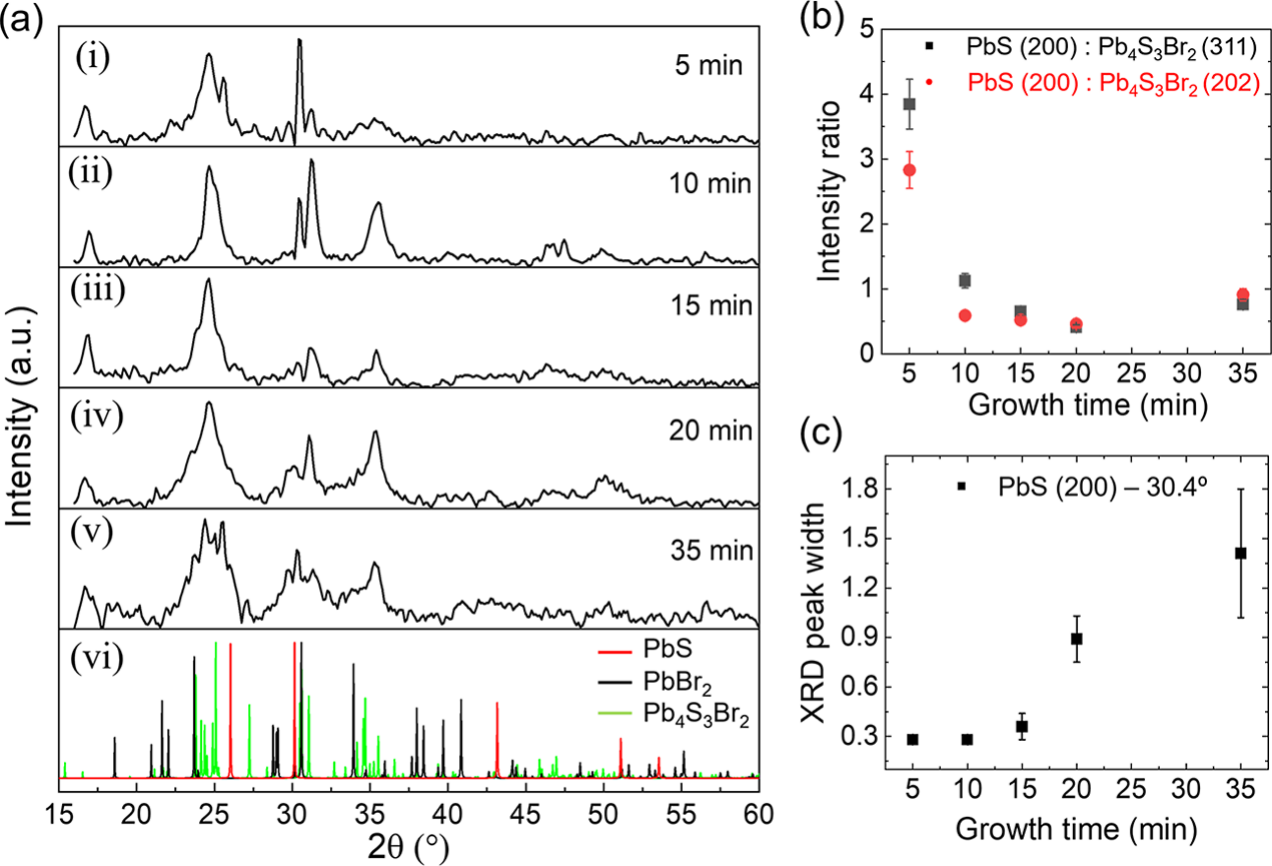
(a) XRD patterns of nanoplatelets synthesized at different growth times: (i) 5 min, (ii) 10 min, (iii) 15 min, (iv) 20 min, and (v) 35 min, along with (vi) simulated XRD patterns of PbS, PbBr_2_, and Pb_4_S_3_Br_2_. (b) Peak intensity ratios of the PbS (200) facet (30.4°) to the Pb_4_S_3_Br_2_ (311) facet (35.2°) and (202) facet (31.1°), extracted from XRD data, showing a gradual decrease of the PbS phase with increasing growth times. (c) Full width at half-maximum (fwhm) of the XRD peak near 30.4° as a function of growth time. The error bars indicate fitting errors (Supporting Information E).

The decrease in the PbS (200) XRD peak intensity indicates the reduction of the PbS composition in the nanoplatelet. Since the basal plane of the core is likely the (200) plane of PbS, we used the width of its corresponding XRD peak to reveal the trend of thickness change. At the nanometer scale, reducing the crystallite size significantly broadens the XRD peaks, as described by the Scherrer equation.^43^ We discovered a clear increasing trend of the XRD peak width with growth time (Figure 4c). It qualitatively reveals the decreasing trend of the PbS thickness by growth time. Nevertheless, the vertically aligned nanoplatelets can narrow down the XRD peaks due to much more crystal periodicities in the lateral dimensions, making it difficult to quantitatively determine the thicknesses (either core or shell) of the nanoplatelets.

To better understand the thickness evolution of the PbS core with growth time, we analyzed its optical absorption and emission spectra. Both absorption and emission peaks blue-shift with increasing growth time. From the photoluminescence-derived photon energies and a quantum confinement model,^42^ the PbS core thicknesses were estimated to be 2.3, 2.0, 1.9, 1.8, and 1.7 nm for growth times of 5, 10, 15, 20, and 35 min, respectively (Supporting Information D). This decreasing trend in core thickness is consistent with the XRD results discussed earlier but contrasts with typical quantum dots or nanosheets, where growth usually leads to red-shifted spectra.^44,45^ TEM analysis (Supporting Information F) shows that the total nanoplatelet thickness remains unchanged with growth time, suggesting an internal crystalline phase transformation. We propose that Br^–^ ions in the reaction solution diffuse into the PbS core, partially converting it into a lead sulfobromide phase and thereby reducing the PbS core thickness. This interpretation is supported by the energy-dispersive X-ray spectroscopy (EDS) result, which shows a higher Br:S ratio at longer growth times (Supporting Information G). These EDS findings are consistent with the XRD data (Figure 4b).

The nanoplatelets exhibit increasing brightness with extended growth. The photoluminescence quantum yield (Supporting Information H) rises from 13% to 22% as the growth time increases from 15 to 35 min (Figure 5d). The photoluminescence e-folding lifetime (τ_e_) shows a marked increase from 5 to 10 min of growth and then stabilizes at approximately 1.2 μs for longer growth times (Figure 5c). These quantum yields and lifetimes are substantially higher and longer than those reported for core-only nanoribbons and nanosheets.^7,42,46,47^ Together, these improvements indicate a significant suppression of crystal defects and surface states, which typically introduces nonradiative decay channels for excitons. We attribute the enhanced optical performance to the inorganic sulfobromide shell, which affords robust passivation; additionally, extended growth likely anneals residual defects, further contributing to the observed optical enhancements.

**Figure 5:**
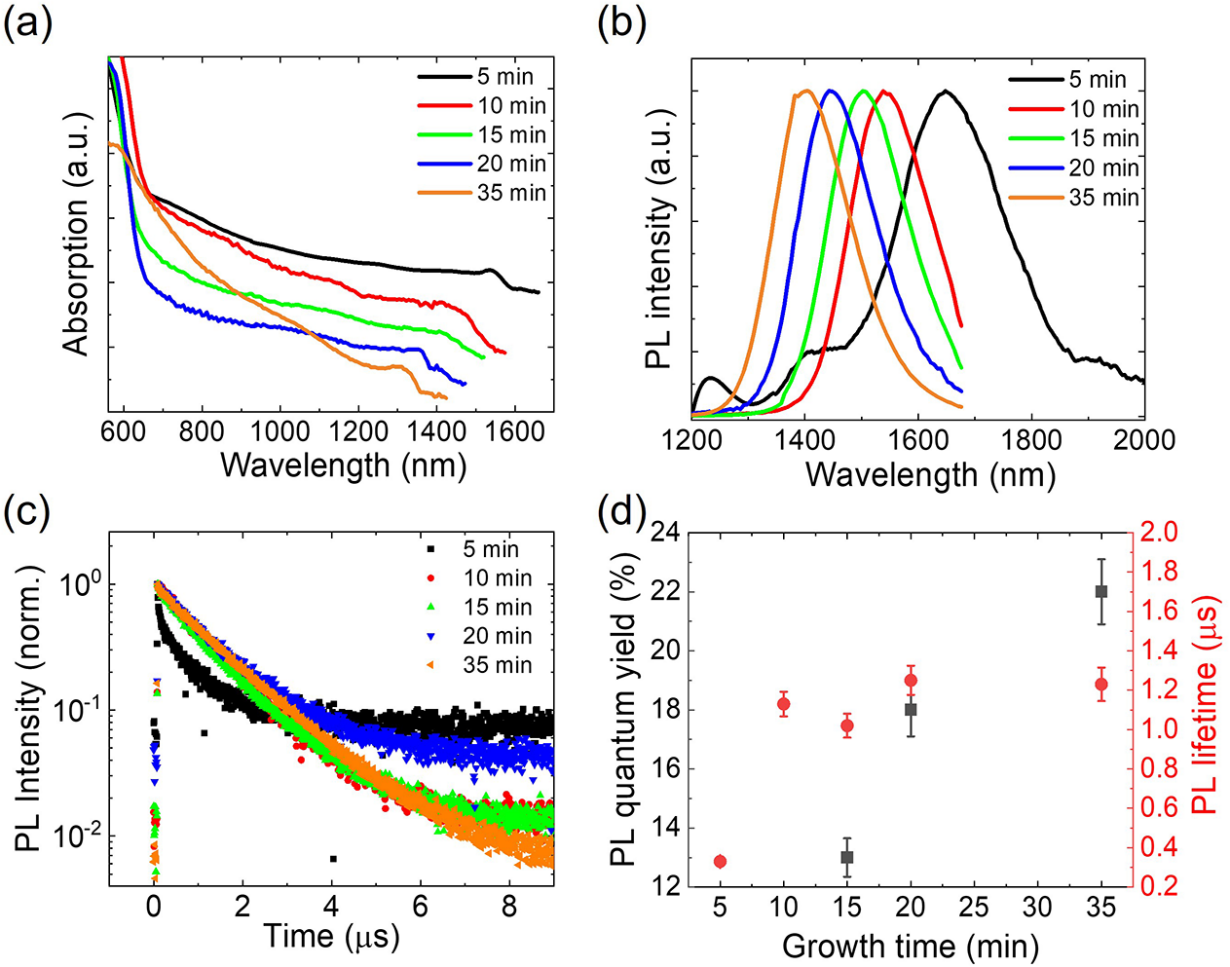
(a) Absorption spectra of PbS nanoplatelets synthesized at 120 °C with different growth times, vertically offset for clarity. (b) Steady-state photoluminescence (PL) spectra of the same samples excited with a continuous-wave 445 nm laser. (c) PL decay traces of nanoplatelets synthesized at different growth times, measured at or near their respective PL peak wavelengths (5 min—1600 nm, 10 min—1538 nm, 15 min—1502 nm, 20 min—1442 nm, 35 min—1406 nm) while excited by a pulsed laser (200 ps pulse duration, 100 kHz repetition rate, 532 nm central wavelength). (d) Growth-time-dependent PL quantum yields (black squares) and lifetimes (red circles). Data for the 5 min and 10 min samples are omitted because a substantial portion of each PL spectrum lies outside the detection range.

A detailed analysis of the photoluminescence dynamics reveals an enhanced radiative decay in nanoplatelets with thinner PbS cores. For samples grown for 15, 20, and 35 min, the photoluminescence quantum yield increases from 13% to 18% and then to 22%, while their photoluminescence e-folding lifetimes remain nearly unchanged (Figure 5d). This trend indicates an increase in the radiative decay rate (*R*_rad_). Fitting the photoluminescence decay traces with a biexponential model yields a submicrosecond fast component τ_1_ and a microsecond slow component τ_2_ (Supporting Information I). τ_1_ decreases significantly as the growth time increases from 10 to 35 min, likely due to stronger exciton binding associated with thinner PbS cores.^48^ The overall photoluminescence decay rate, *R*_PL_ = 1/τ_e_, is the sum of the radiative and nonradiative decay rates, *R*_PL_ = *R*_rad_ + *R*_nrad_. An increase in *R*_rad_ combined with a reduction in *R*_nrad_—attributed to improved passivation of surface states and defects—enhances the quantum yield η, as η = *R*_rad_/(*R*_rad_ + *R*_nrad_), while keeping *R*_PL_ nearly constant (Supporting Information I).

Although a transient blue shift is observed under synthesis conditions, the room-temperature photoluminescence peak remains essentially unchanged during ambient storage: over more than 120 days, we detect no discernible drift in the emission maximum with only occasional minor shifts in a few samples (Supporting Information J). In contrast, the photoluminescence quantum yield gradually increases over time, reaching 35% (Supporting Information J) after 120 days, consistent with a slow self-annealing/passivation process. The e-folding lifetime shows a parallel increase, indicating suppression of nonradiative decay channels typically associated with defect states. The nearly constant full width at half-maximum suggests stable emissive states and uniformity of the nanostructures throughout storage. Taken together, these results demonstrate effective core protection by the inorganic shell, rendering the materials highly resistant to oxidation and aggregation over time and thus well suited for device applications.

The optical properties of the nanoplatelets also depend on the energy level alignment among the core/shell structure. To determine the LUMO (lowest unoccupied molecular orbital) and HOMO (highest occupied molecular orbital) energy levels of each crystalline phase, we used cyclic voltammetry (CV) (Supporting Information K) and optical spectroscopy.

The CV of the nanoplatelets (NPL-1400) exhibits a reduction peak at −0.99 V (Figure 6a­(ii)), which is slightly larger than that of colloidal PbS nanoribbons (NR-1200) emitting at 1200 nm (Figure 6a­(i)). The reduction peak of pure PbBr_2_ powder is −1.37 V (Figure 6a­(iii)). There is a second reduction peak from the nanoplatelets at −1.12 V (Figure 6a­(ii)), which is likely from the lead sulfobromide (Pb_4_S_3_Br_2_) shell. Given the energy level of the Ag/AgNO_3_ reference electrode of −5.12 eV, we obtained the LUMO levels of PbS, Pb_4_S_3_Br_2_, and PbBr_2_ as −4.13, −4.00, and −3.75 eV, respectively. Due to the unreliability of oxidation peaks from PbS nanocrystals, we estimated the HOMO level of the PbS core by subtracting the optical bandgap (0.88 eV) from the LUMO level.^49,50^ This gives an HOMO level of 5.01 eV for the PbS core. For Pb_4_S_3_Br_2_ and PbBr_2_, assuming optical bandgaps of 1.98 eV^35^ and 3.00 eV, respectively,^15^ the HOMO levels are estimated to be −5.98 eV and −6.75 eV, respectively. The LUMO levels of PbBr_2_ and Pb_4_S_3_Br_2_ shells are close to that of the PbS core, relaxing the degree of quantum confinement on electrons. Nevertheless, this core/shell/shell structure still establishes type-I heterojunctions between the PbS core and the Pb_4_S_3_Br_2_ shell, as well as between the Pb_4_S_3_Br_2_ and PbBr_2_ shells (Figure 6b). As a result, excitons are funneled^51,52^ into the lowest-energy state of the PbS core, effectively enhancing the infrared emission from the nanoplatelets.

**Figure 6:**
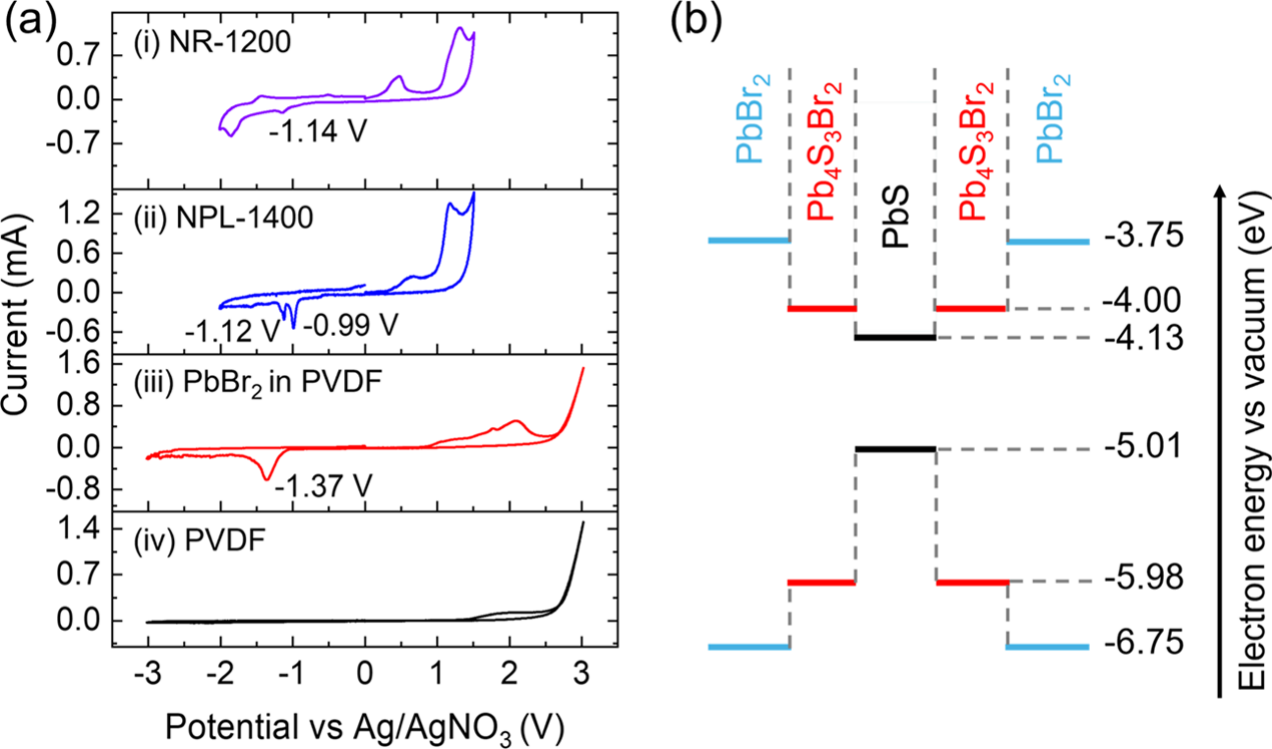
(a) Cyclic voltammetry (CV) measurements of (i) PbS nanoribbons (NR) with photoluminescence (PL) peaked at 1200 nm, (ii) PbS nanoplatelets (NPL) with PL peaked at 1400 nm, (iii) PbBr_2_ in polyvinylidene difluoride (PVDF), and (iv) PVDF only. (b) Energy level diagram (HOMO and LUMO) of the PbS core, Pb_4_S_3_Br_2_ shell, and PbBr_2_ shell, as determined from CV measurements combined with optical spectroscopy.

## Conclusion

In summary, bromide-mediated synthesis yields colloidal PbS nanoplatelets that spontaneously form inorganic core–shell heterostructures with lead sulfobromide and lead bromide shells around a PbS core. This architecture establishes a type-I band alignment, concentrating excitons in the core and thereby enhancing the near-infrared emission. The inorganic shells function as effective passivation layers, suppressing surface defect states and improving photoluminescence quantum yield while also providing ambient stability. Together, these attributes deliver bright, solution-processable near-infrared emitters that are well suited to infrared photonics and optoelectronics, including light sources and detectors where spectral purity and environmental robustness are essential. Looking ahead, controlled tuning of shell composition/thickness and integration with device-relevant matrices and cavities should further refine radiative efficiency and operational stability, advancing these nanoplatelets toward practical, manufacturable components in infrared technologies.

## Experimental Section

### Synthesis

A lead precursor solution was prepared by combining 0.4570 g of PbO (99%, 2.047 mmol), 0.018 g of PbBr_2_ (98%, 0.049 mmol), 10 mL of diphenyl ether (DPE, 63.09 mmol), 1.62 mL of oleic acid (5.04 mmol), and 35 μL of oleylamine (0.106 mmol) in a three-neck flask. The mixture was heated to 120 °C under nitrogen with constant stirring for 2 h until it turned transparent and then degassed for 20 min while maintaining the temperature at 120 °C. Separately, the sulfur precursor was prepared by dissolving 12 mg of thioacetamide (TAA, 0.16 mmol) in a mixture of 70 μL of dimethylformamide (DMF, 0.907 mmol) and 930 μL of trioctylphosphine (TOP, 2.08 mmol) in a three-neck flask. This solution was stirred under nitrogen for 20 min. The sulfur precursor was rapidly injected into the lead precursor solution at 120 °C. After reaction for 20 min, heating was discontinued, and the mixture was allowed to cool naturally to room temperature. The product was purified by adding 10 mL of toluene, shaking vigorously, and centrifuging at 3500 rpm. The washing step was repeated once. The resulting nanoplatelets were redispersed in toluene and stored in the dark.

### Structure Characterization

TEM images were obtained using a Talos L120C G2 electron microscope equipped with a field emission gun operating at 80 kV, while HRTEM images were acquired on a JEOL 3011 microscope. For sample preparation, 10 μL of the colloidal PbS nanoplatelet solution was drop-cast onto a carbon-film-coated copper TEM grid (01840-F, Ted Pella) and dried under vacuum overnight.

X-ray diffraction (XRD) measurements were conducted on a Bruker D8 Advance diffractometer equipped with a Cu tube emitting at 1.54 Å. A high-performance LYNXEYE detector detected the diffracted X-ray signal.

### Optical Spectroscopy

Photoluminescence (PL) measurements were performed using a home-built PL spectroscopy system. A 445 nm continuous-wave laser served as the excitation source. The emitted light was directed into a monochromator (Acton SP-2357, Princeton Instruments) coupled to a femtowatt photoreceiver (model 2153, New Focus Inc.). Time-resolved photoluminescence was detected using a near-infrared photomultiplier tube (950–1700 nm, Hamamatsu H10330–75) mounted on a monochromator (Cornerstone 260, Newport). The excitation source was frequency-doubled pulses at 532 nm generated by an infrared fiber laser (central wavelength of 1064 nm, 100 kHz repetition rate, and 200 ps pulse duration). A custom LabVIEW program was used to record the photoluminescence decay traces.

### Elemental Analysis

The elemental analysis was performed on a Hitachi S-2700, which has an energy-dispersive X-ray spectroscopy (EDX) detector (model PV77–47700-ME) mounted on the instrument. An EDAX Genesis software tool was used to acquire and analyze the spectra for elemental quantification.

### Cyclic Voltammetry

For cyclic voltammetry measurements, a 0.1 M tetrabutylammonium perchlorate (TBAP) solution in acetonitrile was used as the electrolyte. 20 μL of nanoplatelets or nanoribbons were drop-cast onto a glassy carbon working electrode and allowed to dry entirely before measurements. A platinum wire served as the counter electrode. A silver wire in a 0.01 M AgNO_3_ solution in acetonitrile served as the reference electrode. The sweep rate for the measurement was 50 mV/s.

## Supplementary Material



## Notes

Synthesis, transmission electron microscopy, optical spectroscopy, quantum confinement calculation, X-ray diffraction, shape evolution, energy dispersive X-ray spectroscopy, absolute photoluminescence quantum yield, biexponential decay model, optical stability, and cyclic voltammetry (PDF)
