# Supplementary material for: Bright Infrared Colloidal PbS Nanoplatelets with Lead Sulfobromide Shells
Source: Chem Mater. 2026 Jan 19;38(3):1170–7. doi: 10.1021/acs.chemmater.5c02480 (PMC12895391; doi:10.1021/acs.chemmater.5c02480)
Supplement: Supplementary file 1 [file cm5c02480_si_001.pdf]

Supporting Information

Bright Infrared Colloidal PbS Nanoplatelets with Lead Sulfobromide Shells

Sabin Aryal,[1,2] Yiteng Tang,[1,2] Dulanjan Harankahage,[1,2] Mikhail Zamkov,[1,2] and Liangfeng Sun[1,2]*

[1]Department of Physics and Astronomy, Bowling Green State University, Bowling Green, Ohio 43403, USA

[2]Centre for Photochemical Sciences, Bowling Green State University, Bowling Green, Ohio 43403, USA

A. Synthesis

A lead precursor solution was prepared by mixing 0.4570 g of lead oxide (99%, 2.047 mM) and 0.018 g of lead bromide (98%, 0.049 mM) with 10 mL of diphenyl ether (DPE, 63.09 mM), 1.62 mL of oleic acid (5.04 mM), and 35 µL of oleylamine (0.106 mM) in a three-neck flask. The mixture was heated to 120 °C under nitrogen with constant stirring for 2 h until it became transparent. The solution was then degassed for 20 minutes, during which it turned pale yellow. The temperature was subsequently raised and maintained at 120 °C. The sulfur precursor was prepared separately by dissolving 12 mg of thioacetamide (TAA, 0.16 mM) in 70 µL of dimethylformamide (DMF, 0.907 mM) and 930 µL of trioctylphosphine (TOP, 2.08 mM) in a three-neck flask. The solution was stirred under nitrogen for 20 minutes. The sulfur precursor was injected into the lead precursor at 120 °C via syringe. After 20 minutes of reaction, heating was stopped, and the solution was allowed to cool naturally to room temperature. The product was purified by adding 10 mL of toluene, shaking, and centrifuging at 3500 rpm. The washing step

was repeated once, and the resulting nanoplatelets were redispersed in toluene and stored in the dark.

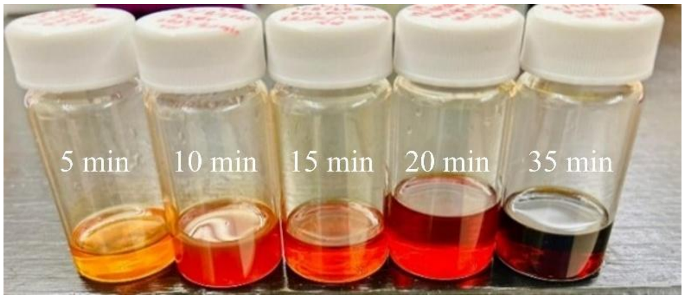

Figure S1. The color of nanoplatelets changes with growth time. With increasing growth times, they turn orange to dark red.

To investigate the effect of lead bromide concentration, we systematically varied the amount of $PbBr_2$ in the reaction solution. The $PbBr_2$ content was increased from 0 g (0 mM) to 0.018 g (0.049 mM), 0.036 g (0.098 mM), and 0.054 g (0.147 mM), while maintaining a constant total lead precursor concentration by proportionally reducing the amount of PbO. All other reagents and reaction conditions were kept identical.

B. Transmission electron microscopy

TEM images were obtained using a Talos L120C G2 electron microscope equipped with a field emission gun operating at 80 kV, while HRTEM images were acquired on a JEOL 3011 microscope. For sample preparation, 10 μL of the colloidal PbS nanoplatelet solution was drop-cast onto a carbon-film–coated copper TEM grid (01840-F, Ted Pella) and dried under vacuum

overnight. Histograms of the nanoplatelet length, width, and thickness, measured from TEM images, are shown in Figure S2.

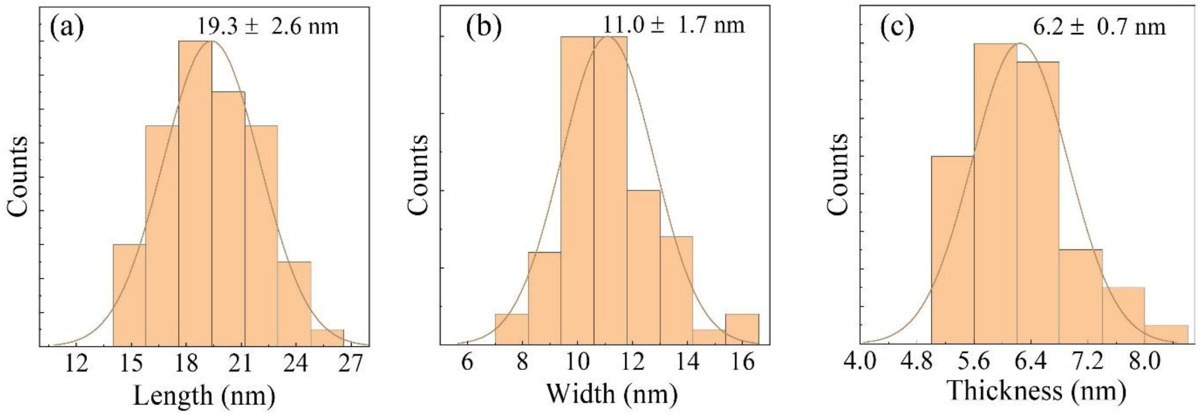

Figure S2. Histograms and fitting curves of the (a) length, (b) width, and (c) thickness of the nanoplatelets (20-minute growth time) measured in their TEM image.

C. Optical spectroscopy

Photoluminescence (PL) measurements were performed using a home-built PL spectroscopy system. A 445 nm continuous-wave laser served as the excitation source. The emitted light was directed into a monochromator (Acton SP-2357, Princeton Instruments) coupled to a femtowatt photoreceiver (Model 2153, New Focus Inc.). A 1100 nm long-pass filter was placed at the monochromator entrance slit to isolate the emission signal from the excitation.

For lifetime measurements, time-resolved photoluminescence was detected using a near-infrared photomultiplier tube (950–1700 nm, Hamamatsu H10330-75) mounted on a monochromator (Cornerstone 260, Newport). The excitation source was frequency-doubled pulses at 532 nm generated from an infrared fiber laser (1064 nm central wavelength, 100 kHz repetition

rate, 200 ps pulse duration). A custom LabVIEW program was used to record the photoluminescence decay traces at or near the peak emission wavelengths of the respective samples.

For sample preparation, the nanoplatelet solution was dried under a nitrogen stream, redispersed in tetrachloroethylene, transferred to a quartz cuvette (Starna Cells, Inc.), and mounted on the sample stage.

D. Quantum confinement calculation

Based on the quantum confinement model developed by Jiang et al,[1] the thicknesses of the nanoplatelets are derived from the photoluminescence (PL) photon energies using the equation:

$$E_{gap} = E_{gap}(\infty) + \frac{1}{1 \cdot 48L - 0.43},$$

where $E_{gap}(\infty)$ is the bulk PbS energy gap, equal to 0.41 eV at room temperature, E_{gap} is the optical energy gap of the nanoplatelet, and L is the thickness of the PbS core in nanoplatelets.

**PL peak position (nm)**	**Photon Energy (eV)**	**Thickness (nm)**
1652	0.75	2.3
1538	0.80	2.0
1502	0.82	1.9

1442	0.86	1.8
1406	0.88	1.7

E. X-ray diffraction

X-ray diffraction (XRD) measurements were conducted on the Bruker D8 Advance diffractometer equipped with a Cu tube emitting at 1.54 Å. The diffracted X-ray signal was detected by a high-performance LYNXEYE detector. Samples were scanned at a diffraction angles (2θ) ranging from 16º to 60º. The data collection time for each XRD measurement is around 50 minutes. The XRD pattern of the lead bromide powder used in the synthesis is shown in Figure S3.

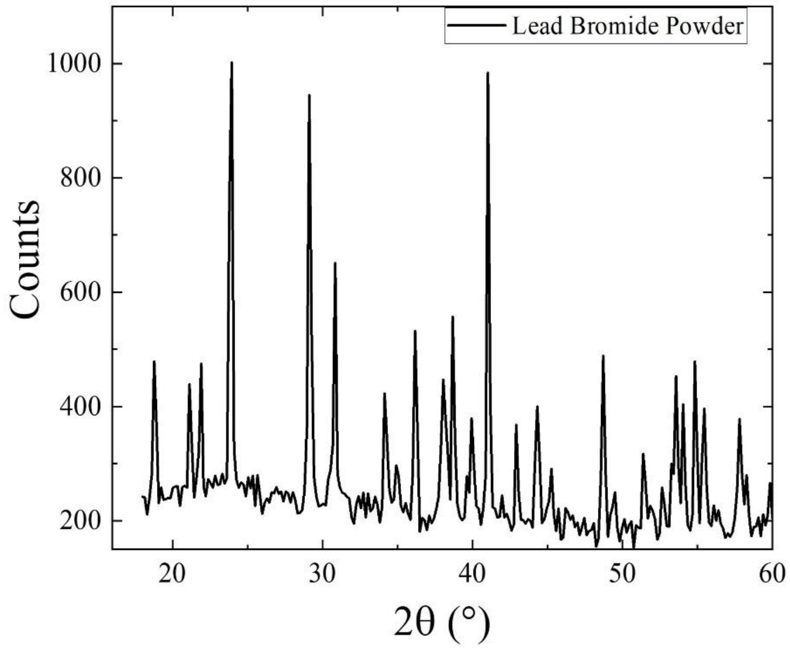

Figure S3. X-ray diffraction of the lead bromide powder (98%) used in synthesizing nanoplatelets.

The fitting results of the two diffraction angles around 31º from different growth-time samples are shown in Figure S4. The XRD angle at 30.4º corresponds to the (200) crystal plane of PbS. Its FWHM increases by growth time.

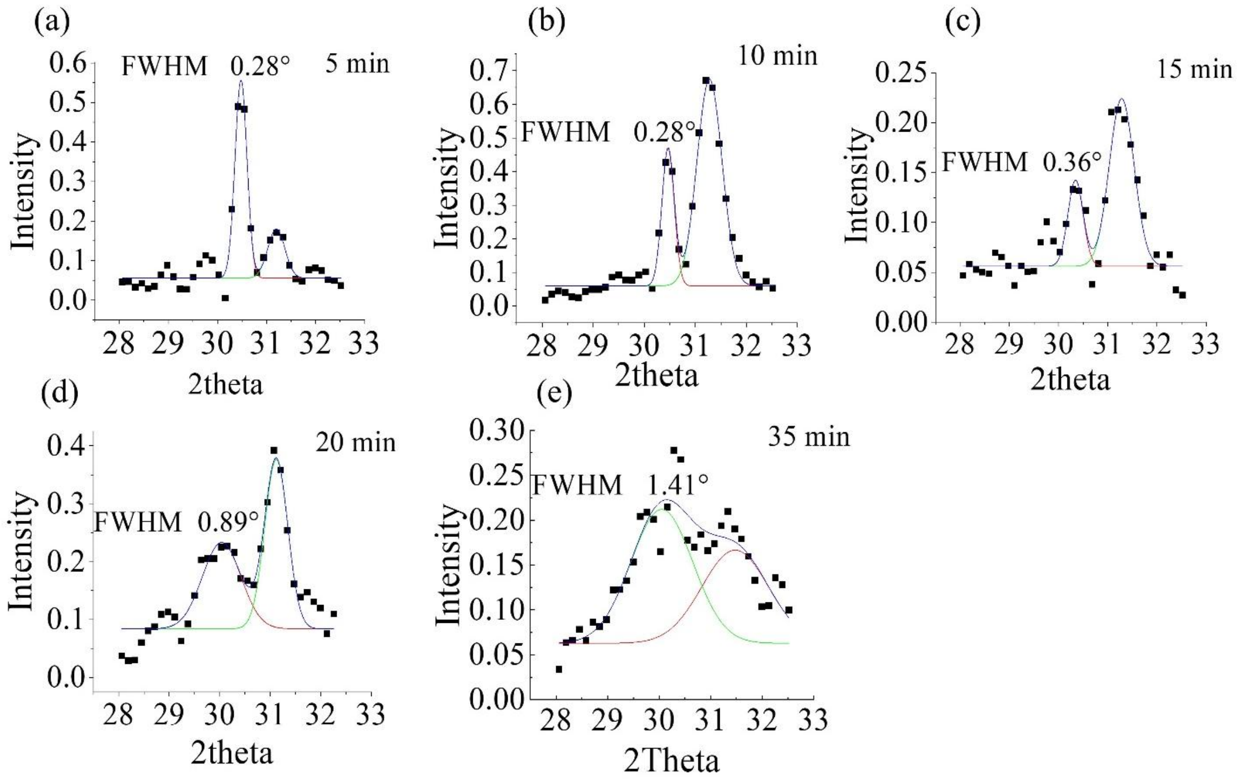

Figure S4. XRD peaks and fitting curves near 31º from the nanoplatelets at different growth time: (a) 5 minutes, (b) 10 minutes, (c) 15 minutes, (d) 20, and (e) 35 minutes.

F. Shape evolution

The nanoplatelets at different growth times were imaged by using Talos electron microscope, shown in Figure S5.

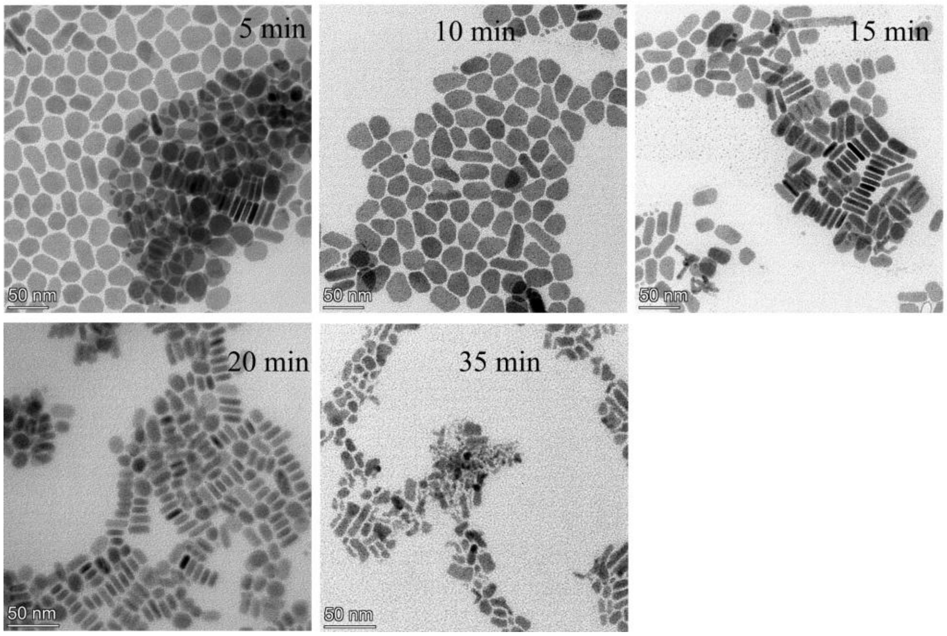

Figure S5. TEM images of nanoplatelets at different growth times.

The thickness of the nanoplatelets is measured from the vertically aligned nanoplatelets in the TEM images. The histograms for the 20-minute and 5-minute samples are shown in Figure S6.

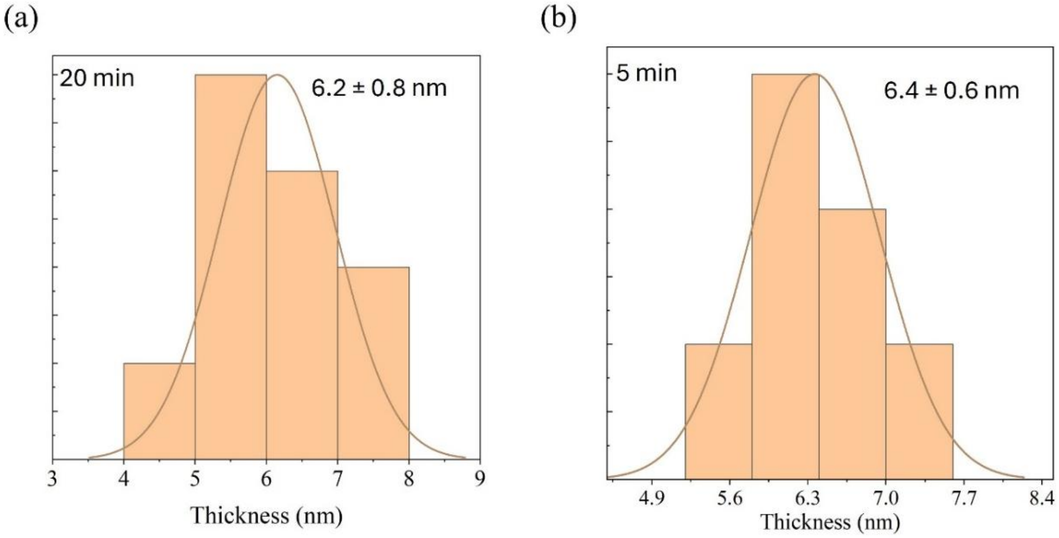

Figure S6. Thickness distributions of the nanoplatelets with (a) 20 minutes (photoluminescence peaked at 1440 nm), and (b) 5 minutes (photoluminescence peaked at 1650 nm) growth time.

G. Energy Dispersive X-ray Spectroscopy

The elemental analysis was done on a Hitachi S-2700. Samples were drop cast on a carbon sticker and allowed to dry overnight in a vacuum. An energy-dispersive X-ray analysis (EDAX) detecting unit (model PV77-47700-ME) is mounted to the instrument. An EDAX Genesis software tool was used to acquire and analyze the spectra for elemental quantification.

We measured the 10-minute and 20-minute samples. The atomic fraction ratio of bromine to sulfur (Br:S) is approximately 2:3 for the 20-minute sample and 1:3 for the 10-minute sample, as shown in Figure S7. These results qualitatively demonstrate an increase in Br composition in the nanoplatelets with longer growth time.

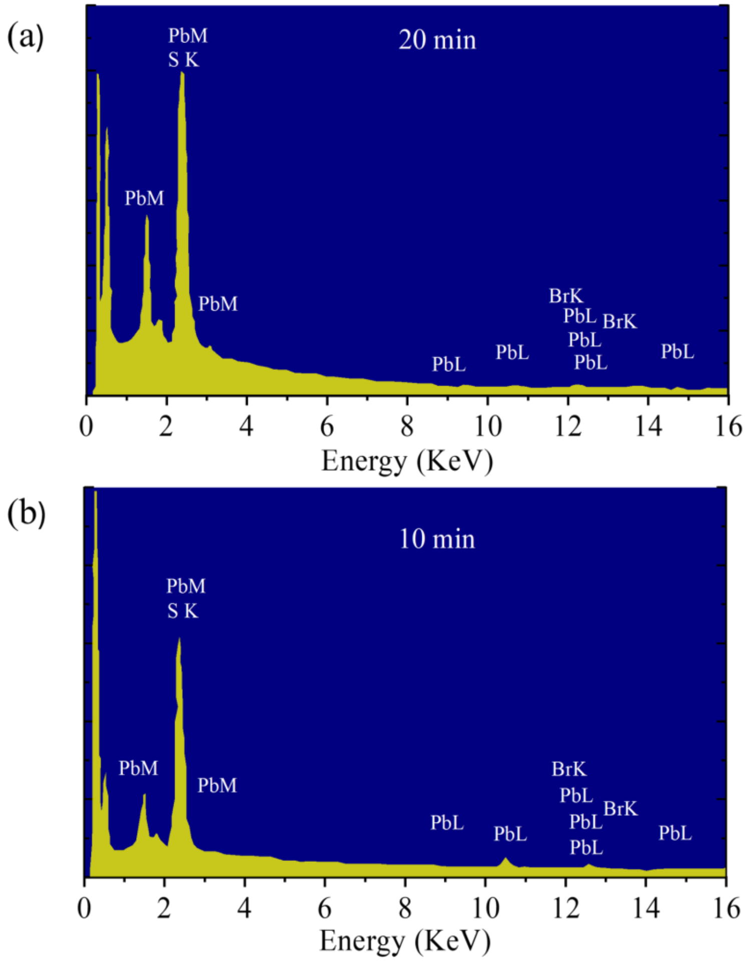

Growth time (min)	Atomic fraction			Ratio (Br:S)
	Pb L	S K	Br K	
20	0.40	0.35	0.25	0.71
10	0.42	0.44	0.14	0.32

Figure S7. Elemental analysis for (a) 20 min and (b) 10 min growth time samples. Bromine contribution for the longer growth time is higher compared to the shorter one.

H. Absolute photoluminescence quantum yield

For photoluminescence quantum yield (PLQY) measurements, an integrating sphere (IS200-4, Thorlabs) was used to diffuse the excitation light and the photoluminescence light from the sample, ensuring isotropic light distribution before detection. It also minimizes the light scattering effect on the quantum yield measurements.[2–4] A quartz–tungsten–halogen lamp (Oriel 66187) served as the excitation source for the sample mounted inside the sphere. The excitation beam was filtered with an 850 nm long-pass filter and a 1000 nm short-pass filter, transmitting light in the 850–1000 nm range. Light exiting the integrating sphere was collected by two lenses and directed to a monochromator (Acton SP-2357, Princeton Instruments) coupled to a femtowatt photoreceiver (Model 2153, New Focus Inc.). A mechanical chopper placed in the beam path, together with a lock-in amplifier, was used to suppress background noise. The spectrum was acquired by recording the photoreceiver signal while scanning the wavelength with the monochromator. The system's spectral response was calibrated using a 20 W quartz–tungsten–halogen lamp (Newport 6319).

A representative measurement for the nanoplatelets is shown in Figure S8. Three spectra were recorded: (a) excitation light only (lamp), (b) sample in the sphere but outside the excitation beam (off), and (c) sample in the excitation beam (on). For each photon-number spectrum (intensity × wavelength vs. wavelength), five parameters were determined: L_a, L_b, L_c (areas under the excitation spectra), and P_b, P_c (areas under the PL spectra), where the subscripts a, b, and c correspond to the three conditions above. The PLQY was then calculated using the following equation:[2]

$$\eta = \frac{(L_b * P_c) - (L_c * P_b)}{L_a(L_b - L_c)}$$

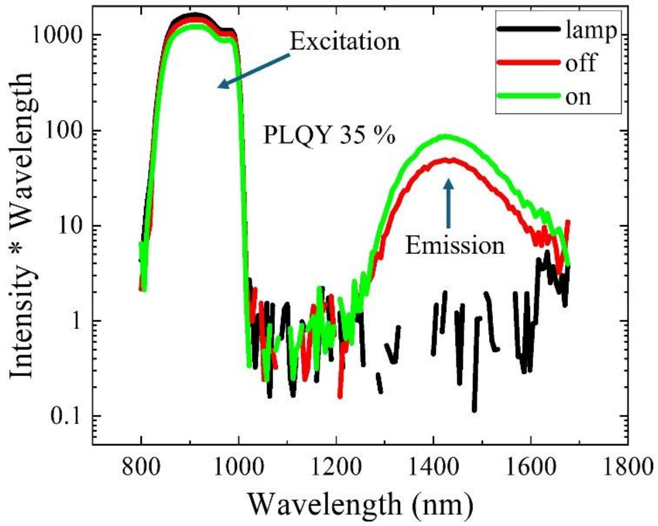

Figure S8. PLQY of aged PbS nanoplatelets synthesized at 120 °C with a growth time of 35 minutes.

I. Bi-exponential decay model

We used a bi-exponential decay model to fit each photoluminescence decay trace (Figure S9). The extracted fast decay constants (τ_1) and slow decay constants (τ_2) are listed in Table S1, together with the corresponding photoluminescence quantum yields (PLQYs) and e-folding lifetimes.

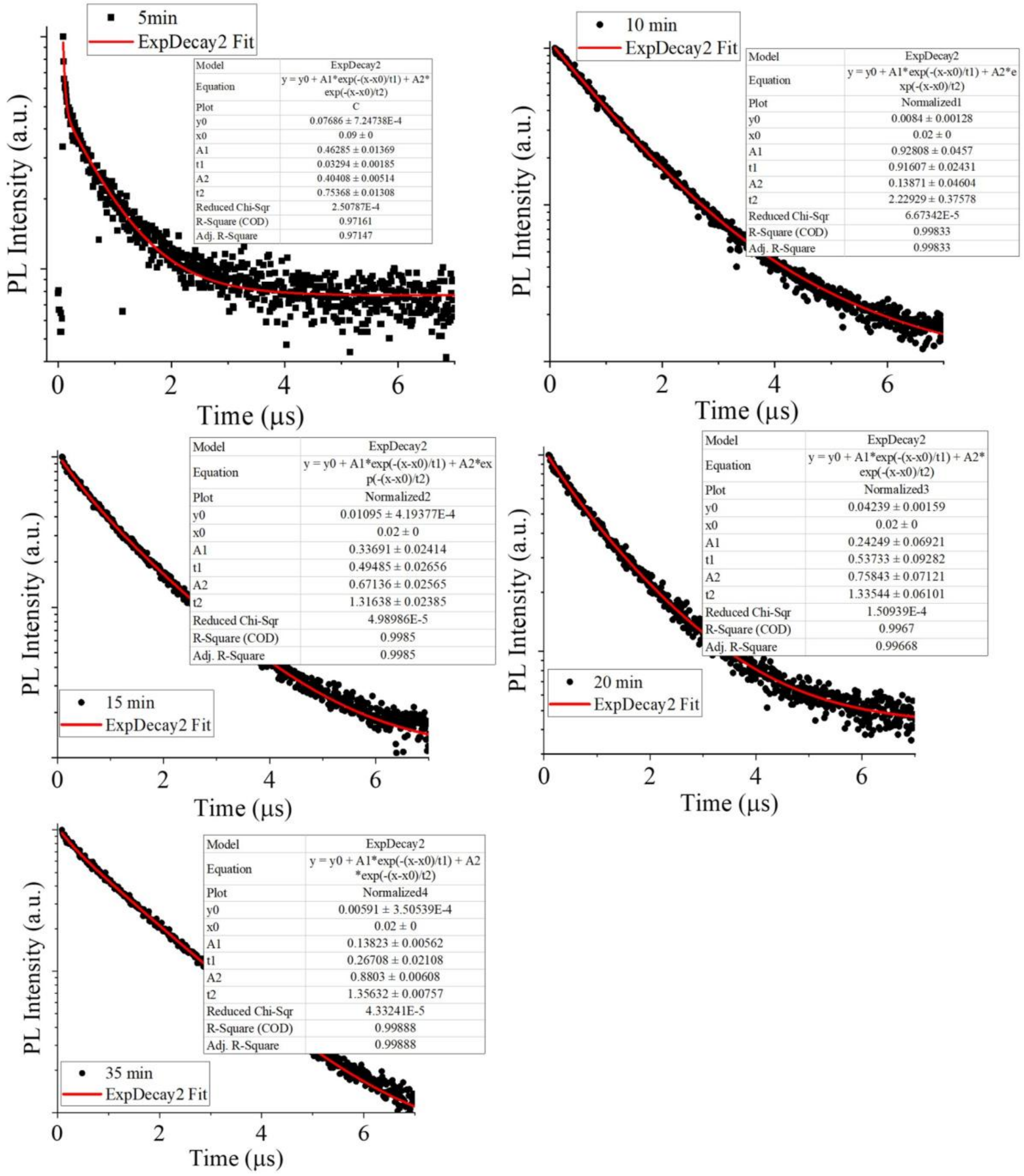

Figure S9. Photoluminescence decay traces and corresponding bi-exponential fitting curves for nanoplatelets synthesized at different growth times.

Table S1. Photoluminescence quantum yield, e-folding lifetime (τe), fast decay constants (τ_1) and slow decay constants (τ_2) of the nanoplates at different growth times.

Growth time (min)	PLQY (%)	τ_e (μs)	τ_1 (μs)	τ_2 (μs)
5	N/A	0.33	0.03	0.75
10	N/A	1.13	0.91	2.22
15	13	1.02	0.49	1.31
20	18	1.25	0.53	1.33
35	22	1.23	0.26	1.35

J. Optical stability

We performed spectroscopy measurements on the as-synthesized nanoplatelets. They were then stored in toluene in the dark at room temperature for 120 days, after which the measurements were repeated for comparison. All nanoplatelet samples synthesized with different growth times exhibited increases in both photoluminescence lifetime (Figure S10a) and quantum yield (Figure S10b) after aging. The photoluminescence peak wavelength and the ratio of full width at half maximum (FWHM) to peak wavelength remain nearly unchanged between each pair of as-synthesized and aged samples (Figure S10c, d).

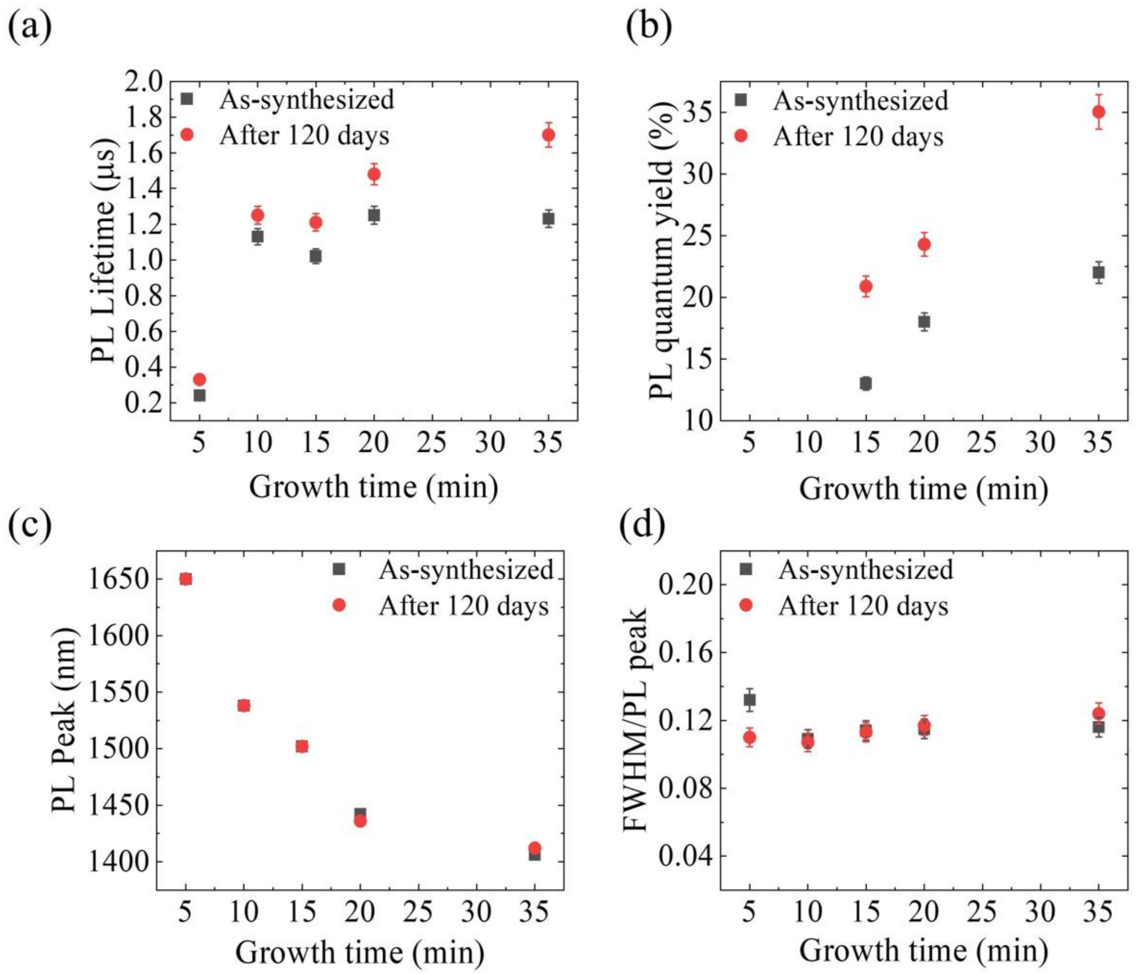

Figure S10. Comparison of (a) photoluminescence (PL) lifetime, (b) PL quantum yield, (c) PL peak wavelength, and (d) the ratio of FWHM to PL peak between the as-synthesized nanoplatelets (squares) and aged nanoplatelets (circles) at their respective growth times.

K. Cyclic voltammetry

For cyclic voltammetry measurements, a 0.1 M tetrabutylammonium perchlorate (TBAP) solution in acetonitrile was prepared as the electrolyte solution. 20 µL of nanoplatelets or nanoribbons were drop-casted on the glassy carbon working electrode and allowed to dry completely before taking

measurements. A platinum wire serves as the counter electrode. A silver wire in 0.01 M AgNO3 acetonitrile solution serves as the reference electrode. Cyclic voltammetry measurements were followed by 20 minutes of nitrogen purging directly to the electrolyte in the electrochemical cell to make the entire system oxygen-free. At the time of measurement, the nitrogen supply tube is not in contact with the electrolyte, but still inside the electrochemical cell with a continuous flow of nitrogen.

For the CV measurement of lead bromide powder, we mixed lead bromide powder (0.060 g) with polyvinylidene fluoride (PVDF) (0.012 g) in the ratio 5:1 by weight, dissolved it in 1-methyl-2-pyrrolidinone (3 mL), and followed it by sonication. 20 μL of this solution is drop-casted on the working electrode and allowed to dry completely. For the PVDF-only measurement, 0.025 g of PVDF is dissolved in 2.5 mL of 1-methyl-2-pyrrolidinone. The solution is vortexed and sonicated afterward to make a uniform solution. 20 μL of this solution is drop-cast on the working electrode and dried overnight under vacuum to make the solvent evaporate completely. The sweep rate for the measurement is 50 mV/s. All the measurements were done at room temperature.

References:

(1) Jiang, Z.; Tang, Y.; Antu, A. D.; Premathilaka, S. M.; Cayer, M. L.; Heckman, C. A.; Moroz, P.; Zamkov, M.; Sun, L. Colloidal Nanoribbons: From Infrared to Visible. *J Phys Chem Lett* **2022**, *13* (39), 8987–8992. https://doi.org/10.1021/acs.jpclett.2c02390.

(2) de Mello, J. C.; Wittmann, H. F.; Friend, R. H. An Improved Experimental Determination of External Photoluminescence Quantum Efficiency. *Advanced Materials* **1997**, *9* (3), 230–232. https://doi.org/10.1002/adma.19970090308.

(3) Bhandari, G. B.; Subedi, K.; He, Y.; Jiang, Z.; Leopold, M.; Reilly, N.; Lu, H. P.; Zayak, A. T.; Sun, L. Thickness-Controlled Synthesis of Colloidal PbS Nanosheets and Their Thickness-Dependent Energy Gaps. *Chemistry of Materials* **2014**, *26* (19), 5433–5436. https://doi.org/10.1021/cm502524z.

(4) Antu, A. D.; Jiang, Z.; Premathilka, S. M.; Tang, Y.; Hu, J.; Roy, A.; Sun, L. Bright Colloidal PbS Nanoribbons. *Chemistry of Materials* **2018**, *30* (11), 3697–3703. https://doi.org/10.1021/acs.chemmater.8b00467.
